# Intraperitoneal Chemotherapy Strategies in Pancreatic Ductal Adenocarcinoma: A Systematic Review of Hyperthermic Intraperitoneal Chemotherapy, Normothermic Intraperitoneal Chemotherapy, and Pressurized Intraperitoneal Aerosol Chemotherapy

**DOI:** 10.3390/cancers18020182

**Published:** 2026-01-06

**Authors:** Nency Ganatra, Ahmed Abdelhakeem, Pragya Jain, Saivaishnavi Kamatham, Dina Elantably, Oluwatayo Adeoye, Hani M. Babiker, Conor D. O’Donnell, Umair Majeed

**Affiliations:** 1 Department of Internal Medicine, Baptist Hospitals of Southeast Texas, Beaumont, TX 77701, USA; nencykganatra@gmail.com (N.G.);; 2 Division of Haematology and Oncology, Department of Internal Medicine, Mayo Clinic, Jacksonville, FL 32224, USA

**Keywords:** pancreatic cancer, HIPEC, PIPAC, NIPEC paclitaxel

## Abstract

Background: Peritoneal metastasis represents an aggressive disease pattern in pancreatic ductal adenocarcinoma (PDAC), traditionally associated with poor survival and limited therapeutic options. Emerging intraperitoneal chemotherapy strategies—including hyperthermic intraperitoneal chemotherapy (HIPEC), normothermic intraperitoneal paclitaxel (NIPEC/IP-PTX), and pressurized intraperitoneal aerosol chemotherapy (PIPAC)—have been investigated to improve local tumor control and survival outcomes. Methods: We systematically reviewed published studies evaluating HIPEC, NIPEC/IP-PTX, and PIPAC in PDAC, including adjuvant, cytoreductive, and palliative settings. Study characteristics, feasibility, perioperative outcomes, oncologic outcomes, and risk of bias were analyzed. Results: Across modalities, intraperitoneal treatment strategies demonstrated acceptable feasibility and safety profiles in appropriately selected patients. Adjuvant HIPEC following pancreatectomy showed reduced local–regional recurrence signals in limited cohorts. CRS + HIPEC among patients with isolated peritoneal metastases yielded encouraging multi-year survival in highly selected candidates achieving complete cytoreduction. NIPEC/IP-PTX demonstrated favorable ascites control, symptom relief, and potential conversion to resection in select patients. PIPAC was primarily used in unresectable, heavily pretreated, palliative peritoneal metastasis settings, with goals centered on disease stabilization, histologic regression, and symptom control rather than curative intent. Conclusions: Intraperitoneal chemotherapy strategies in PDAC appear feasible with signals of meaningful clinical benefit in select settings. While CRS + HIPEC may benefit carefully selected metastatic patients, NIPEC/IP-PTX and PIPAC hold value primarily in symptom control and disease stabilization. Larger prospective trials are needed to define patient selection, optimize treatment protocols, and clarify survival benefit.

## Simple Summary

Pancreatic cancer often spreads to the lining of the abdomen, leading to severe symptoms and very limited treatment options. Standard chemotherapy does not reach this area well, which is one reason outcomes remain poor. This review explores treatments that deliver cancer medicines directly into the abdominal cavity, allowing much higher local drug levels with fewer whole-body side effects. These approaches include heated chemotherapy during surgery, repeated liquid chemotherapy through a small port, and aerosolized chemotherapy given with minimally invasive procedures. By examining the safety, benefits, and limitations of these methods, our goal is to understand which patients may benefit most and how these treatments could be combined with standard care. Our findings may help guide future research, improve patient selection, and support the development of clinical trials focused on better options for patients with abdominal spread of pancreatic cancer.

## 1. Introduction

Pancreatic ductal adenocarcinoma (PDAC) is regarded as one of the most lethal malignancies worldwide, often characterized by rapid progression, early dissemination, and resistance to systemic therapy. Globally, PDAC consistently ranks among the top causes of cancer-related mortality and is projected to become the second leading cause of cancer-related death within 2030 [1,2]. Despite advances in surgical technique, perioperative management, and systemic therapies, which include multi-agent chemotherapy regimens such as FOLFIRINOX and gemcitabine-nab-paclitaxel, the prognosis for most patients remains guarded, with 5-year survival rates below 12% [3]. Even in patients undergoing curative-intent pancreatectomy, recurrence rates exceed 70%, and peritoneal dissemination constitutes one of the earliest and most common failure patterns, likely due to the anatomic proximity of the pancreas to the peritoneal cavity and the biological propensity of PDAC for peritoneal spread [4,5].

The unique tumor microenvironment (TME) of PDAC plays a central role in therapeutic resistance, metastasis, and intraperitoneal seeding. Dense desmoplastic stroma, hypovascularity, aberrant extracellular matrix remodeling and immunosuppressive cellular infiltrates, including regulatory T cells, tumor-associated macrophages, and myeloid-derived suppressor cells, collectively contribute to poor drug delivery, immune evasion, and treatment failure [6,7,8]. Traditional systemic chemotherapies achieve limited penetration into the peritoneal cavity due to physiologic barriers, elevated intraperitoneal pressure, and impaired microvascular perfusion, resulting in subtherapeutic concentrations at sites of microscopic peritoneal dissemination [9]. Consequently, patients with peritoneal metastases (PM) experience significantly worse outcomes than those with other metastatic patterns, with median survival historically ranging 3–6 months on systemic chemotherapy alone [10].

Given these therapeutic limitations, there is renewed interest in compartment-directed approaches that deliver antineoplastic agents directly into the peritoneal cavity to enhance locoregional cytotoxicity. Intraperitoneal (IP) therapies, including hyperthermic intraperitoneal chemotherapy (HIPEC), normothermic intraperitoneal chemotherapy such as IP paclitaxel (NIPEC/IP-PTX), and pressurized intraperitoneal aerosol chemotherapy (PIPAC), can overcome the pharmacokinetic and microenvironmental obstacles that limit systemic therapy effectiveness in peritoneal disease. By exploiting the peritoneal-plasma barrier, IP therapy achieves markedly higher local drug concentrations with reduced systemic exposure, thereby enabling intensified locoregional treatment while maintaining acceptable toxicity [11,12,13]. These modalities have garnered significant interest in PDAC, where peritoneal recurrence after resection and peritoneal metastasis at diagnosis are particularly prevalent and clinically consequential.

HIPEC, delivered immediately following cytoreductive surgery (CRS) or in the adjuvant setting after pancreatectomy, combines heated chemotherapy with direct peritoneal perfusion. Hyperthermia enhances drug penetration, impairs DNA repair, induces tumor cell apoptosis, and disrupts stromal barriers, thereby augmenting chemotherapeutic efficacy [14,15,16]. Although HIPEC is well-established in other peritoneal surface malignancies such as pseudomyxoma peritonei and colorectal or ovarian carcinomatosis, its application in PDAC has historically been limited due to concerns about toxicity, postoperative complications, and the aggressive biology of the disease [17]. However, emerging intraperitoneal chemotherapy experience in gastrointestinal and peritoneal surface malignancies, including PDAC, has demonstrated encouraging safety, feasibility, and oncologic signals in carefully selected patients [11,12,13,17].

NIPEC/IP-PTX represents a complementary strategy that leverages the unique pharmacokinetic properties of paclitaxel, a highly lipophilic, high-molecular-weight agent that achieves exceptionally high peritoneal-to-plasma ratios and prolonged locoregional exposure [18,19,20]. Early Japanese and international studies have shown that IP paclitaxel, when combined with systemic chemotherapy (e.g., S-1, gemcitabine, or nab-paclitaxel), can induce radiologic regression of peritoneal disease, improve ascites control, promote conversion-to-resection, and extend survival beyond the historically expected outcomes for PDAC with PM [21,22,23]. These findings suggest that NIPEC/IP-PTX may meaningfully alter disease trajectory in a subset of patients.

PIPAC, a recent innovation in intraperitoneal therapy, utilizes pressurized aerosolization of chemotherapy delivered laparoscopically to achieve uniform peritoneal distribution and enhanced tissue penetration. Preclinical and early clinical studies demonstrate that pressure gradients facilitate deeper drug infiltration into tumor nodules, while aerosolized droplets improve spatial coverage and pharmacodynamic effect [24,25,26,27]. PIPAC offers several theoretical advantages compared with liquid intraperitoneal chemotherapy, including repeatability, low systemic exposure, and the ability to obtain real-time histologic assessment using the Peritoneal Regression Grading Score (PRGS). Although PIPAC experience in PDAC is still emerging, early reports suggest that it may provide symptom control and measurable biological activity in selected patients, with an acceptable safety profile [24,25,26,27].

Despite these advances, the literature on intraperitoneal therapies in PDAC remains heterogeneous, fragmented, and constrained by limited sample sizes. Study designs vary widely, ranging from single-canter retrospective series to prospective phase I/II studies and one randomized controlled trial (RCT). Additionally, patient selection criteria, IP regimens, chemotherapy doses, perfusion temperatures, device platforms, and clinical endpoints differ substantially across studies [11,12,13,14,17]. This variability complicates direct comparisons and limits the ability to draw definitive conclusions, highlighting the need for rigorous systematic synthesis.

Accordingly, this systematic review aims to consolidate and critically appraise the current evidence on intraperitoneal therapy strategies for PDAC. The primary objective is to evaluate the feasibility, safety, perioperative outcomes, pharmacologic rationale, and oncologic efficacy of hyperthermic intraperitoneal chemotherapy (HIPEC), normothermic intraperitoneal paclitaxel (NIPEC/IP-PTX), and pressurized intraperitoneal aerosol chemotherapy (PIPAC). Secondary objectives include comparing patient-selection frameworks across modalities, assessing risk of bias and quality of evidence, and identifying knowledge gaps to guide future trial design and clinical translation. By integrating data from sixteen prospective and retrospective studies, this review provides a comprehensive and clinically focused evaluation of the evolving role of intraperitoneal therapies in PDAC.

## 2. Materials and Methods

### 2.1. Eligibility Criteria

Studies were eligible for inclusion if they evaluated intraperitoneal (IP) therapies specifically hyperthermic intraperitoneal chemotherapy (HIPEC), normothermic intraperitoneal chemotherapy with paclitaxel (NIPEC/IP-PTX), or pressurized intraperitoneal aerosol chemotherapy (PIPAC) in adult patients (≥18 years) with histologically confirmed pancreatic ductal adenocarcinoma (PDAC). Eligible designs included prospective clinical trials, retrospective cohorts, pilot studies, and single-arm feasibility studies that reported extractable data on feasibility, perioperative morbidity, 30-day mortality, locoregional recurrence (LRR), overall survival (OS), progression-free survival (PFS) where applicable, conversion-to-resection, radiologic response, histologic response by peritoneal regression grading score (PRGS), or other clinically relevant oncologic outcomes. Both resectable and metastatic settings were included when IP therapy was administered either at the time of curative-intent surgery, during cytoreductive surgery (CRS) for peritoneal metastases, or as palliative or disease-controlling therapy. We excluded studies involving non-PDAC primaries unless PDAC outcomes were separately extractable; laboratory-based, preclinical, or translational studies without clinical outcome reporting; conference abstracts lacking full data; review articles; case reports; letters or commentaries; studies without extractable IP-related outcomes; and articles published in languages other than English. Studies evaluating CRS alone without HIPEC, systemic-only chemotherapy, or radiation-only interventions were also excluded unless they served as comparator arms within a study primarily assessing IP therapy. In cases of overlapping cohorts from the same institution, the most comprehensive or recent dataset was selected, and earlier overlapping reports were excluded to avoid duplication of patient data [28,29].

### 2.2. Information Sources

A systematic search was conducted across PubMed/MEDLINE, Embase, Scopus, and the Cochrane Library, covering all literature from database inception through August 2025. These databases were selected to ensure comprehensive coverage of surgical oncology, gastrointestinal oncology, pharmacology, and peritoneal surface malignancy literature. To maximize the inclusiveness of the search and ensure capture of all relevant IP therapy studies in PDAC, the reference lists of included articles were manually reviewed for additional eligible studies. ClinicalTrials.gov and the WHO International Clinical Trials Registry Platform (ICTRP) were screened to identify ongoing or unpublished trials, particularly those evaluating HIPEC protocols, NIPEC paclitaxel regimens, or PIPAC dose-escalation studies [30,31]. No limitations were imposed on geographic origin or institution. Only full-text peer-reviewed publications were considered. Abstracts without complete data, preprints without final peer review, and grey literature (e.g., theses or unpublished registries) were excluded due to insufficient methodological transparency.

### 2.3. Search Strategy and Study Selection

This systematic review was conducted in accordance with the Preferred Reporting Items for Systematic Reviews and Meta-Analyses (PRISMA) 2020 guidelines. A completed PRISMA checklist is included in the Supplementary Materials, and the study selection process is depicted in the PRISMA flow diagram (Table S1). The protocol was not prospectively registered; however, the objectives, eligibility criteria, data extraction strategy, and analytic framework were defined a priori. Retrospective PROSPERO registration was explored but was not pursued as manuscript development was already substantially completed. Search terms were constructed to reflect three major domains: PDAC, peritoneal metastasis or peritoneal dissemination, and IP therapy modalities. Boolean operators connected the following key terms: “pancreatic ductal adenocarcinoma,” “PDAC,” “pancreatic cancer,” “peritoneal metastasis,” “peritoneal carcinomatosis,” “intraperitoneal chemotherapy,” “hyperthermic intraperitoneal chemotherapy,” “HIPEC,” “intraperitoneal paclitaxel,” “IP-PTX,” “normothermic intraperitoneal chemotherapy,” “PIPAC,” “pressurized intraperitoneal aerosol chemotherapy,” “aerosolized chemotherapy,” and “cytoreductive surgery.” MeSH terms and EMTREE subject headings were incorporated where appropriate to improve specificity. After deduplication, titles and abstracts underwent screening by two independent reviewers using predefined eligibility criteria. Articles deemed potentially relevant were retrieved for full-text review. Any disagreements regarding eligibility were resolved through consensus discussion or adjudication by a third reviewer. Full-text screening included verification of study design, patient population, PDAC specificity, IP regimen, and availability of extractable outcomes such as OS, LRR, conversion-to-resection, PRGS, or morbidity. The Preferred Reporting Items for Systematic Reviews and Meta-Analyses (PRISMA) guidelines informed the overall study selection workflow [32,33].

### 2.4. Data Collection Process

Data extraction followed a structured and piloted protocol aligned with PRISMA recommendations. Two reviewers independently extracted key study characteristics, including first author, publication year, study design, patient setting (resected PDAC, isolated peritoneal metastasis, cytology-positive disease), sample size, IP regimen (drug, dose, carrier solution, perfusate temperature for HIPEC, pressure settings for PIPAC, dwell time, cycle frequency), surgical details (extent of CRS, CC score, presence of pancreatectomy), and perioperative management elements. Oncologic outcomes included OS, LRR, radiologic response, histologic regression, PRGS categories, ascites control, and conversion-to-resection. Feasibility endpoints (e.g., successful port placement, completion rates for IP cycles), safety outcomes (e.g., postoperative pancreatic fistula, hematologic toxicity, Grade ≥ 3 complications), and 30-day mortality were also extracted. Discrepancies in extraction were resolved through discussion. When data were unclear, Supplementary Materials, appendices, or institutional protocol descriptions were reviewed. No unpublished data were solicited from study authors. Survival outcomes were extracted as reported; no reconstruction of Kaplan–Meier curves or derivation of effect estimates was performed due to the systematic review (non-meta-analytic) design [34,35].

### 2.5. Risk of Bias Assessment

Risk of bias was evaluated using modified domains from the Newcastle–Ottawa Scale (NOS) for observational studies and methodological criteria from phase I/II trial evaluation frameworks. Domains included selection bias (eligibility clarity, representativeness of cohort, handling of consecutive patients), comparability (matching or adjustment for clinical covariates such as age, ECOG performance status, extent of peritoneal metastasis, or systemic therapy backbone), and outcome assessment (completeness of follow-up, definition of recurrence, standardized PRGS scoring for PIPAC, and clarity of postoperative morbidity classification using Clavien–Dindo). Studies with unclear reporting or incomplete follow-up were coded as “unclear risk,” and those with strong prospective methodology and standardized endpoints were designated “low risk.” No study met criteria for “high risk” across all domains, but several retrospective cohorts exhibited inherent limitations due to nonrandomized design and potential selection bias favoring fitter surgical candidates [36,37,38].

### 2.6. Synthesis Methods

Due to profound clinical and methodological heterogeneity, including differences in patient selection (resected vs. metastatic disease; biologically favorable vs. unselected cohorts), treatment strategies (adjuvant HIPEC, CRS + HIPEC, NIPEC, PIPAC), chemotherapy regimens, endpoints (OS defined from different time points), varying reporting of PCI/PRGS, and lack of comparable control groups in most studies. Under these conditions, pooled statistical synthesis, meta-analysis was not pursued. Instead, a narrative synthesis approach was employed. Studies were grouped by modality: adjuvant HIPEC, CRS + HIPEC for peritoneal metastases, NIPEC/IP-PTX, and PIPAC. Within each category, we compared feasibility, perioperative morbidity, IP regimen characteristics, and oncologic outcomes. We emphasized within-group consistency, cross-study patterns, and mechanistic plausibility informed by the broader PDAC and peritoneal pharmacology literature. Due to heterogeneity in study populations, dosing parameters, perfusion methods, and clinical endpoints, formal statistical tests for heterogeneity were not applicable. Similarly, survival outcomes were not standardized across studies due to differing definitions (e.g., from pancreatectomy vs. from PM diagnosis vs. from IP initiation). These constraints precluded the use of pooled effect measures. Instead, results were synthesized using structured narrative comparison, highlighting areas of consensus (e.g., reproducibility of OS in NIPEC/IP-PTX cohorts), modality-specific strengths (e.g., LRR reduction in adjuvant HIPEC), and limitations (e.g., early discontinuation in PIPAC). Mechanistic and translational literature was integrated to contextualize observed clinical patterns [39,40,41].

### 2.7. Evaluation of Publication Bias

Evaluating publication bias in a purely narrative systematic review requires indirect assessment using consistency of reporting, presence of negative studies, and alignment between expected and published outcomes. No formal funnel plot analyses were performed because no quantitative pooling was conducted. Instead, we assessed for potential publication bias by considering: (1) the presence of prospective phase I/II studies reporting feasibility concerns; (2) inclusion of small or unfavorable retrospective cohorts; (3) balance in reporting of adverse events; and (4) identification of unpublished or ongoing trials with incomplete dissemination of results [42,43]. Although early-phase IP therapy research often favors publication of promising feasibility or survival outcomes, multiple included studies reported modest efficacy or high attrition (particularly in PIPAC), suggesting that major publication bias is unlikely to fully account for observed patterns. Nevertheless, the preponderance of small, single-institution studies introduces intrinsic vulnerability to selective reporting.

## 3. Results

### 3.1. Study Selection

**Figure 1:**
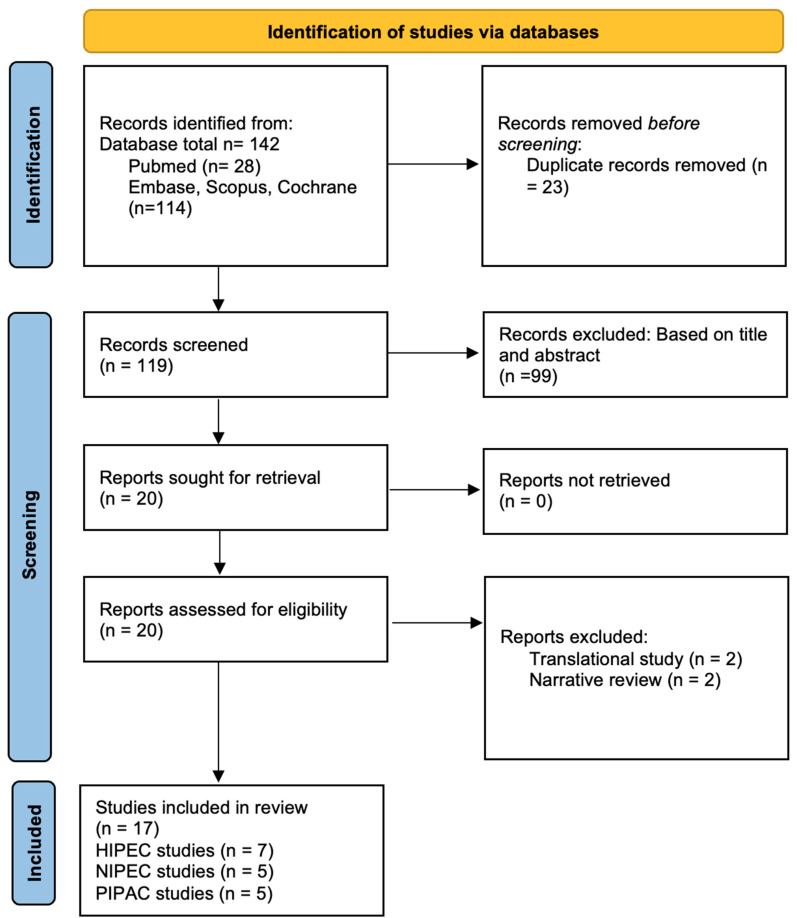
PRISMA 2020 flow diagram illustrating the study identification, screening, eligibility assessment, and inclusion process for this systematic review.

The initial search across PubMed and Google Scholar identified a total of 142 records as shown in Figure 1. After automated and manual duplicate removal (n = 23), 119 unique titles and abstracts underwent screening. Most exclusions at this stage were due to studies unrelated to pancreatic ductal adenocarcinoma (PDAC), absence of an intraperitoneal (IP) therapeutic component, or reporting limited to systemic therapy alone without a dedicated regional treatment strategy. Following title and abstract screening, 19 articles were retrieved for full-text assessment based on apparent relevance to intraperitoneal modalities, including hyperthermic intraperitoneal chemotherapy (HIPEC), normothermic intraperitoneal paclitaxel (NIPEC/IP-PTX), and pressurized intraperitoneal aerosol chemotherapy (PIPAC).

Among the 20 full-text articles evaluated, 3 were excluded. Two focused exclusively on preclinical or translational IP delivery systems without human subjects and therefore did not meet eligibility criteria requiring extractable clinical feasibility, safety, or oncologic outcomes. One additional article was excluded because it constituted a narrative review or overlapping systematic review without unique patient-level data relevant to PDAC. Ultimately, 17 studies met the full eligibility criteria and were included in the qualitative synthesis. These comprised 7 studies evaluating HIPEC (either in the adjuvant setting after pancreatectomy or as part of cytoreductive surgery for isolated peritoneal metastases) [44,45,46,47,48,49,50], 5 studies examining NIPEC/IP-PTX administered with systemic therapy [51,52,53,54,55], and 5 studies reporting outcomes from PIPAC programs utilizing oxaliplatin or cisplatin/doxorubicin [56,57,58,59,60]. Collectively, the included studies represented a diverse spectrum of intraperitoneal therapeutic strategies, ranging from early-phase feasibility trials to retrospective multi-institutional analyses, reflecting the current breadth of clinical experience with regional treatment approaches for PDAC.

### 3.2. Study Characteristics

The characteristics of the included studies, including design, sample size, and treatment modality, are summarized in Table 1.

The seventeen included studies represented heterogeneous geographical, methodological, and therapeutic approaches to intraperitoneal management of PDAC. Study designs encompassed prospective phase I/II trials [44,45,51,52,53,54,55,59], retrospective institutional cohorts [48,49,50,56,57,58,60], matched comparative analyses [48], and one randomized controlled trial [45] Sample sizes ranged from small pilot cohorts of 10–14 patients to larger multicenter phase II NIPEC studies involving more than 40 participants. For PIPAC studies by Horvath et al. and Kurtz et al., [56,57] total cohort sizes were reported but PDAC-specific sample sizes were not separately provided; therefore, these studies were included qualitatively without assigning modality-specific N values.

In accordance with the narrative style of the reference article, a conceptual study characteristics table as described above summarizes key variables including study design, geographic setting, treatment modality (HIPEC vs. NIPEC vs. PIPAC), IP chemotherapy agent(s), perfusion temperature (for HIPEC), pressure settings (for PIPAC), cycle frequency, peritoneal metastasis status, CRS completeness cytoreduction score (CC-0/1 vs. CC-2/3), survival endpoints. 

#### 3.2.1. HIPEC Studies and Treatment Characteristics

HIPEC studies (n = 7) included two evaluating adjuvant HIPEC following pancreatectomy (Yurttas 2021; Padilla-Valverde 2024) [44,45], one perioperative HIPEC pilot (Padilla-Valverde 2021) [46], and four assessing CRS + HIPEC in patients with isolated peritoneal metastases (Grotz 2023; Gudmundsdottir 2023; Yan 2024; Tentes 2018) [47,48,49,50].

An earlier prospective exploratory study of adjuvant HIPEC following pancreatectomy was reported by Tentes et al., demonstrated reduced local–regional recurrence and survival signals in highly selected patients. However, this cohort predates modern systemic therapy standards and lacks contemporary methodological rigor; therefore, while acknowledged for historical relevance, it was not included in the primary evidence synthesis, which focuses on contemporary standardized HIPEC protocols [61]. Across all modalities, the studies collectively included more than 250 patients with PDAC receiving intraperitoneal therapy, although the exact number varied due to mixed-primary cohorts in some PIPAC series. Perfusion drugs varied by modality: gemcitabine was primarily used in adjuvant and perioperative HIPEC; cisplatin/mitomycin combinations predominated in CRS + HIPEC protocols; paclitaxel was universal in NIPEC/IP-PTX regimens; and oxaliplatin or cisplatin-doxorubicin combinations were used for PIPAC.

#### 3.2.2. NIPEC/IP Paclitaxel Programs

NIPEC/IP-PTX studies (*n* = 5) primarily originated from Japanese and East Asian cohorts using standardized regimens of IP paclitaxel combined with systemic chemotherapy (S-1, gemcitabine, nab-paclitaxel). These included the multicenter Phase II Satoi 2017 trial, a single-institution retrospective comparison (Satoi 2017 JHBP), the Takahara 2014 phase II study, the Yamada 2020 phase I/II GnP/IP-PTX trial, and the Takahara 2021 dose-establishing phase I study [51,52,53,54,55].

#### 3.2.3. PIPAC Programs and Efficacy Evidence

PIPAC programs (*n* = 5) comprised both prospective feasibility investigations and retrospective analyses of repeated PIPAC cycles using oxaliplatin or cisplatin-doxorubicin combinations. PIPAC programs were predominantly applied in unresectable, heavily pretreated, and palliative peritoneal metastasis settings, with primary objectives including disease stabilization, histologic regression, symptom control, and quality-of-life improvement rather than curative intent. Histologic response using PRGS and radiologic RECIST stability formed the main efficacy endpoints [56,57,58,59,60].

### 3.3. Risk of Bias

Risk of bias for observational and interventional studies was assessed using the Newcastle–Ottawa Scale, with scores across selection, comparability, and outcome domains summarized in Table 2.

Risk of bias for observational studies was evaluated using the Newcastle–Ottawa Scale, which assesses three domains: study selection (0–4 points), comparability (0–2 points), and outcome assessment (0–3 points), with a maximum score of 9 indicating lowest risk of bias. Risk-of-bias assessment revealed the inherent methodological constraints of early-phase surgical and interventional oncology studies. Prospective phase I/II trials exhibited generally low selection bias, clearly defined eligibility criteria, standardized toxicity reporting, and prospective data collection. However, limited sample sizes and absence of control arms restricted interpretability.

Retrospective studies demonstrated moderate risk of bias due to selection of surgically fit candidates, institutional variations in CRS extent, and incomplete adjustment for confounders such as ECOG status, systemic therapy backbone, or interval from peritoneal metastasis diagnosis to intervention. In Gudmundsdottir 2023, use of a matched control chemotherapy group partially mitigated confounding but did not eliminate inherent selection differences [48].

Outcome assessment bias was minimized in prospective NIPEC and PIPAC programs that utilized standardized PRGS scoring or objective endpoints such as ascites volume, conversion surgery eligibility, and completion of prespecified IP cycles. However, definitions of morbidity varied across studies, particularly regarding Clavien–Dindo classifications for postoperative HIPEC programs [62].

### 3.4. Results of Individual Studies

Key clinical outcomes from the included studies, including feasibility, short-term safety, pathological or radiological response, conversion to resection, and overall survival, are summarized in Table 3.

Only studies reporting extractable clinical outcomes including feasibility, perioperative morbidity, conversion-to-resection, PRGS response, or median OS were included in this summary table; early-phase dose-finding trials and pilot safety studies without survival endpoints were excluded.

#### 3.4.1. HIPEC Studies

Adjuvant HIPEC after pancreatectomy: Yurttas et al. (2021) [44] conducted a prospective phase I/II single-arm study of adjuvant gemcitabine HIPEC (1000 mg/m^2^ at 42 °C for 60 min) in resected PDAC without peritoneal metastasis. Treatment feasibility exceeded 85%, 30-day mortality was 0%, postoperative pancreatic fistula (POPF) occurred in 3 of 13 at-risk patients, and exploratory median overall survival (mOS) reached 16.1 months, with 1-year OS of 62.5% [56]. Padilla-Valverde et al. (2024) [45] performed a randomized controlled trial comparing adjuvant HIPEC (gemcitabine 120 mg/m^2^ at 41–42 °C with CO_2_ agitation) to standard pancreatectomy without HIPEC. The HIPEC arm exhibited significantly reduced locoregional recurrence (LRR): 10% vs. 52%; *p* = 0.022 without excess perioperative complications, demonstrating the first controlled evidence supporting adjuvant HIPEC in PDAC. In this trial, median DFS was longer in the HIPEC arm compared with standard resection (14 vs. 10 months), while median OS remained similar between groups (17.1 vs. 18.0 months), indicating that the primary benefit of adjuvant HIPEC lies in improved locoregional disease control rather than early survival prolongation.

Perioperative HIPEC program: Padilla-Valverde et al. (2021) [46] reported an initial pilot experience of gemcitabine-based HIPEC performed at the time of pancreatectomy in 10 patients, demonstrating procedural feasibility and a perioperative safety profile comparable to standard resection and rationale for a full trial integrating both locoregional approaches.

CRS + HIPEC for isolated peritoneal metastasis: Grotz et al. (2023) [47] presented prospective pilot data showing that in patients with isolated peritoneal metastases who responded to ≥6 months of systemic therapy, CRS + HIPEC (cisplatin and mitomycin C) achieved median OS of ~26 months and high CC-0/1 cytoreduction rates, with acceptable morbidity. Gudmundsdottir et al. (2023) [48] reported a retrospective matched cohort analysis demonstrating mOS of 41 months for CRS + HIPEC compared to 19 months with chemotherapy alone. Although limited by retrospective design, findings suggested substantial survival advantage in selected patients. Yan et al. (2024) [49] described a single-center series of 10 patients undergoing CRS + HIPEC with median OS of 24.2 months and no perioperative mortality, reinforcing safety and feasibility. Tentes et al. (2018) [50] provided additional retrospective support for feasibility but limited extractable outcomes. Across CRS + HIPEC studies, 30-day mortality ranged 0–4.3%, and major morbidity ranged 20–43%, comparable to CRS programs for other peritoneal surface malignancies.

#### 3.4.2. NIPEC/IP Paclitaxel Studies

NIPEC/IP-PTX programs demonstrated remarkable consistency in survival outcomes across heterogeneous study populations. The multicenter Satoi et al. (2017 Ann Surg) [51] phase II trial enrolling 33 patients with PDAC peritoneal metastases or positive cytology reported median survival time (MST) of 16.3 months, 1-year OS of 62%, and conversion-to-resection in 24% of patients a substantial improvement over historical MST of 3–6 months. A paired retrospective cohort from the same group (Satoi 2017 JHBPS) [52] demonstrated MST of 20 months vs. 10 months for controls, improved ascites control, and increased conversion rates with IP-PTX. Takahara et al. (2014) [53] demonstrated feasibility and early signals of efficacy in 10 patients with gemcitabine-refractory PDAC with malignant ascites receiving IP-PTX with S-1. Yamada et al. (2020) [54] evaluated IP-PTX with gemcitabine and nab-paclitaxel (GnP) in a phase I/II design, reporting MST of 14.5 months and conversion surgery in 17%. Takahara et al. (2021) [55] established feasible dosing for IP-PTX combined with GnP in a phase I study. Collectively, NIPEC/IP-PTX studies demonstrate reproducible MST of ~14–20 months, reliable ascites control, 1-year OS ~60%, and conversion surgery rates 17–24%, with manageable hematologic toxicity and device-related adverse events.

#### 3.4.3. PIPAC Studies

Horvath et al. (2018) [56] evaluated PIPAC with cisplatin-doxorubicin (CD) or oxaliplatin (OX), reporting mOS ~12.7 months in PDAC subsets and low-grade adverse events predominating. Kurtz et al. (2018) [57] assessed feasibility and safety of outpatient PIPAC-OX (OPC2 protocol), demonstrating low serious adverse event rates and utility for staging and quality-of-life maintenance. Di Giorgio et al. (2020) [58] analyzed 14 PDAC cases across two centers, reporting mOS 9.7 months and 50% histologic regression by PRGS, with only one intraoperative perforation across 45 PIPAC procedures. Kim et al. (2021) [59] identified oxaliplatin 120 mg/m^2^ as the recommended phase II dose through a dose-escalation trial, noting peritoneal response and RECIST stability in 62.5% after the first cycle. Khosrawipour et al. (2017) [60] reported a single-center series of 20 patients with peritoneal carcinomatosis from pancreatic adenocarcinoma treated with salvage PIPAC using cisplatin 7.5 mg/m^2^ and doxorubicin 1.5 mg/m^2^ at 6-week intervals after progression on, or intolerance to, at least one line of systemic chemotherapy. Consistent with its current clinical role, PIPAC in PDAC is largely employed as a palliative intervention in unresectable peritoneal metastasis, aiming to stabilize disease, induce histological regression, relieve ascites, and support quality of life rather than achieve cure. The mean number of PIPAC cycles was 2.1 (range 1–4), and objective histologic tumor regression was observed in 7 of 20 patients (35%), including complete regression in 2 (10%). Median overall survival was 36.6 weeks (~9 months) from the first PIPAC application. Toxicity was favorable, with predominantly CTCAE grade 1–2 events, no CTCAE grade 3–4 toxicities, and one treatment-related death from small bowel obstruction (2.4%) Across all PIPAC programs, median OS ranged 9–13 months, PRGS improvements occurred in 50–60%, and major toxicity was low, although early discontinuation due to progression. Comparative characteristics of available intraperitoneal treatment strategies, including ideal patient selection, key advantages, limitations, safety considerations, and strength of evidence, are summarized in Table 4.

### 3.5. Additional/Contemporary Investigations

Ongoing and recent clinical investigations are expanding the evidence base for intraperitoneal chemotherapy approaches, supported by advances in pharmacologic understanding and intraperitoneal drug-delivery strategies [62]. A multicenter phase I trial by Choi et al. subsequently evaluated nanoliposomal irinotecan (nal-IRI) administered during cytoreductive surgery combined with hyperthermic intraperitoneal chemotherapy, demonstrating feasibility and acceptable safety [63]. Advanced drug delivery strategies are being explored, with Silvia Breusa et al. highlighting the potential of combining drug-loaded nanocarriers with PIPAC delivery systems [64].

Pharmacokinetic Optimization: Parallel efforts focus on pharmacokinetic optimization through albumin-bound formulations, with A. di Giorgio et al. investigating nabpaclitaxel-PIPAC protocols and N. Lang et al. examining nab-paclitaxel and cisplatin combinations administered via pressurized intraperitoneal aerosol chemotherapy [65,66].

Patient Selection and Biomarkers: Future trials aim to validate patient selection algorithms, with F. Koumpa et al. emphasize the critical importance of translational biomarkers for diagnostic, prognostic, and therapeutic deployment in peritoneal metastases management [67].

Translational Research: Translational research continues to inform mechanistic understanding, as evidenced by R. Sundar et al., who demonstrated enhanced T-cell infiltration and immune/stromal changes in peritoneal tumors following PIPAC treatment, providing insights into the biological mechanisms underlying locoregional therapy effectiveness [68].

## 4. Discussion

This systematic review synthesizes contemporary evidence for intraperitoneal chemotherapy strategies in pancreatic ductal adenocarcinoma (PDAC), spanning adjuvant hyperthermic intraperitoneal chemotherapy (HIPEC), cytoreductive surgery plus HIPEC (CRS + HIPEC) for peritoneal metastases (PM), normothermic intraperitoneal paclitaxel (NIPEC/IP-PTX), and pressurized intraperitoneal aerosol chemotherapy (PIPAC). Across 16 clinical series, intraperitoneal (IP) approaches were consistently feasible, with peri-treatment morbidity and mortality comparable to major pancreatic or peritoneal surface surgery, and they produced survival outcomes that appear to exceed historical expectations for PDAC patients with peritoneal involvement treated with systemic therapy alone [3,10,18,44,45,46,47,48,49,50,51,52,53,54,61,68,69,70]. Our findings support a growing view that, in carefully selected patients, PDAC with peritoneal dissemination may be amenable to compartment-directed strategies that augment the effects of systemic therapy rather than representing an invariably terminal systemic stage [10,18,44,48,70,71,72,73].

### 4.1. Positioning Intraperitoneal Therapy Against Systemic-Only Benchmarks

Historically, PDAC patients with metastatic disease have had a median overall survival (mOS) in the range of 3–6 months without effective chemotherapy and 8–11 months with contemporary systemic regimens such as FOLFIRINOX or gemcitabine-based combinations [3,10,14,18,69,70]. Even within metastatic cohorts, peritoneal involvement has been associated with particularly poor outcomes, reflecting both advanced tumor biology and challenges in achieving adequate drug exposure across the peritoneal surface [5,9,10,14,40,72]. Recent population-based and institutional analyses confirm that the peritoneum is among the most common and prognostically adverse metastatic sites in PDAC, with survival often shorter than for patients with isolated lung or distant nodal metastases [4,5,14,70,73]. Against this backdrop, the survival ranges observed in NIPEC/IP-PTX (mOS ~14–20 months), CRS + HIPEC for low-volume peritoneal disease (mOS ~24–41 months), and PIPAC programs (mOS ~9–13 months) appear clinically meaningful [44,45,46,47,48,49,50,51,52,53,54,55,56,57,58,59,61,68,69]. These outcomes are broadly consistent with recent systematic reviews and narrative syntheses of IP therapy in PDAC, which report mOS values in the 12–24-month range for well-selected patients, often with improved ascites control and a non-trivial proportion achieving secondary resection [69,73]. Furthermore, data from large GI oncology series in gastric and colorectal peritoneal metastases, including the recent meta-analysis by Boshier et al. demonstrating a pooled mOS of 16.4 months and a survival advantage for combined IP plus systemic therapy over systemic therapy alone, provide cross-disease support for the paradigm that regional intensification can translate into tangible clinical benefit when applied judiciously [12,13,17,24,71].

### 4.2. Adjuvant HIPEC: Reducing Locoregional Recurrence Without Compromising Safety

In the adjuvant setting, Yurttas et al. and Padilla-Valverde et al. collectively demonstrate that intraperitoneal gemcitabine HIPEC at the time of pancreatectomy is technically feasible, with 30-day mortality of 0–5% and postoperative complication profiles comparable to standard pancreatectomy [44,45,46]. The randomized trial by Padilla-Valverde et al. is particularly noteworthy as the first controlled evidence suggesting that adjuvant HIPEC can significantly reduce locoregional recurrence (LRR) without increasing perioperative morbidity [45]. This aligns with prior pharmacologic data showing that intraperitoneal gemcitabine achieves high peritoneal-to-plasma ratios with acceptable systemic exposure and may be safely delivered in the immediate postoperative period [9,11,12,21,68,73].

Nonetheless, the impact of adjuvant HIPEC on distant metastatic patterns and long-term survival remains less clear. Both Yurttas et al. and Padilla-Valverde et al. report mOS values in the mid-teen range (approximately 16–18 months), which are not dramatically superior to modern adjuvant systemic-only benchmarks, suggesting that while peritoneal control improves, hematogenous and nodal dissemination continue to drive late failure [3,4,44,45,69,70,71]. This observation dovetails with contemporary mechanistic work highlighting that the PDAC metastatic cascade to the peritoneum shares molecular mediators with distant metastasis (e.g., EMT pathways, integrins, chemokine axes), such that purely regional intensification may be insufficient without optimized systemic strategies [6,7,8,39,40,71]. Integrating HIPEC with intensified perioperative systemic regimens and more refined biological selection (e.g., unfavorable transcriptomic or ctDNA profiles) may be necessary to convert LRR reduction into durable survival gains.

### 4.3. CRS + HIPEC for Isolated Peritoneal Metastases: A “Regional Oligometastatic” State?

For patients with low-volume PM who have responded to ≥6 months of systemic therapy, CRS + HIPEC emerges as the most aggressive but also the most promising strategy in terms of survival, with multiple series reporting mOS exceeding two years and some approaching 3–4 years [47,48,49,50,64,69,73]. Grotz et al. and Gudmundsdottir et al. demonstrate that, when complete or near-complete cytoreduction (CC-0/1) is achieved, outcomes approach those seen in CRS + HIPEC programs for colorectal peritoneal metastases, with acceptable major morbidity (20–43%) and very low perioperative mortality (0–4.3%) [17,48,49,64,71,73]. Yan and Tentes further support the notion that, in highly selected PDAC patients with isolated PM, CRS + HIPEC can yield meaningful long-term survival, although sample sizes remain small and subject to referral and selection biases [49,50,64,69].

These findings align with larger peritoneal surface oncology literature in which PM is increasingly conceptualized as a spectrum from diffuse systemic dissemination to a “regional oligometastatic” state amenable to aggressive locoregional therapies [12,13,17,70,72]. Avula et al. highlight that peritoneal metastasis in PDAC is orchestrated by specific molecular programs—such as ECM remodeling, integrin signaling, and mesothelial-mesenchymal interactions—that may be particularly sensitive to high local drug exposure achieved by HIPEC, even when systemic disease burden is otherwise controlled [6,39,40,72]. In this context, CRS + HIPEC may be viewed not as a rescue procedure for end-stage disease but as part of a multimodal, biology-driven strategy in patients who demonstrate chemo-sensitive disease, limited peritoneal carcinomatosis index (PCI), and preserved performance status [17,44,47,48,70,73].

However, important caveats remain. First, all CRS + HIPEC series are non-randomized and subject to substantial selection bias, with younger, fitter patients and those with favorable systemic response disproportionately represented [47,48,49,50,64,69,73]. Second, the contribution of HIPEC beyond maximal CRS alone cannot be disentangled in most studies, and extrapolation from colorectal or gastric data may not fully account for PDAC’s unique biology [12,13,17,71]. Third, standardized criteria for PCI thresholds, systemic therapy duration, and resectability are lacking, limiting generalizability and making it difficult to define a reproducible “CRS + HIPEC pathway” in PDAC [17,44,70,73]. Accordingly, multi-year survival observed in CRS + HIPEC cohorts likely reflects a combination of favorable tumor biology, durable systemic chemotherapy response, and aggressive surgical selection, rather than a direct causal effect of HIPEC in isolation. Prospective, multi-institutional registries using harmonized PCI scoring, PRGS assessment for microscopic residual disease, and detailed systemic therapy annotation will be crucial to refine these indications.

### 4.4. NIPEC/IP-PTX: Consistent Disease Control and Meaningful Conversion-to-Resection

NIPEC/IP-PTX regimens stand out for their reproducible survival, ascites control, and conversion-to-resection rates across independent Japanese and East Asian cohorts [51,52,53,54,61]. The multicenter phase II trial by Satoi et al. reported mOS of 16.3 months, 1-year OS of 62%, and conversion surgery in 24% of patients with peritoneal metastasis or positive cytology—figures echoed by the single-center retrospective study and by Takahara and Yamada in gemcitabine/nab-paclitaxel (GnP)-based programs [51,52,53,54,61]. These outcomes substantially exceed historical expectations for PDAC PM managed with systemic therapy alone and approximate the survival observed in some metastatic PDAC populations without peritoneal involvement [3,10,18,51,52,53,54,55,69,70,71].

Mechanistically, paclitaxel’s high molecular weight and lipophilicity underpin peritoneal-to-plasma concentration ratios of >1000:1 with prolonged peritoneal residence, enabling repeated high-intensity locoregional exposure at doses that remain tolerable systemically [18,19,20,23,51,52,53,54,55,56,69,73]. Avula et al. and others emphasize that adhesive interactions between tumor cells and the mesothelial ECM, along with stromal barrier effects, are key steps in the peritoneal metastatic cascade; repeated IP-PTX cycles may disrupt these adhesive and stromal interactions over time, thereby not only controlling macroscopic ascites but also eroding microscopic peritoneal deposits [6,39,40,72,73]. The relatively high conversion-to-resection rates observed suggest that NIPEC/IP-PTX may act as a cytoreductive “bridge” enabling secondary pancreatectomy and, in select cases, even subsequent CRS + HIPEC.

From a practical standpoint, NIPEC/IP-PTX demands durable intraperitoneal access and carries risks of port-related complications, infection, and catheter failure, although most reported adverse events are manageable and primarily hematologic [51,52,53,54,61,69,73]. Device-related issues and treatment fatigue contribute to early discontinuation in some patients, underscoring the importance of patient counseling and supportive care. As with CRS + HIPEC, it is also important to recognize that reported survival advantages with NIPEC/IP-PTX largely arise from highly selected patients with preserved performance status, treatment-responsive disease, and favorable tumor biology, and therefore should not be interpreted as a direct therapeutic effect of intraperitoneal therapy alone. There is also a need to harmonize IP-PTX regimens—doses, cycle intervals, and systemic combinations—across centers, as highlighted by Safari et al. and Brind’Amour et al., who note substantial heterogeneity in protocols and outcomes across published PDAC IP series [69,73]. Future trials may explore bi-directional approaches combining NIPEC/IP-PTX with PIPAC or perioperative HIPEC, but such strategies will require careful attention to cumulative toxicity and quality-of-life outcomes.

### 4.5. PIPAC: A Repeatable, Biologically Active Option for Frailer Patients

PIPAC offers a minimally invasive, repeatable platform for delivering chemotherapy as a pressurized aerosol, with the potential advantages of uniform peritoneal distribution, improved tissue penetration, and low systemic exposure [24,25,26,27,56,57,58,59,60]. Across PDAC-focused PIPAC programs by Horvath, Di Giorgio, Kim, and others, completion rates for at least two PIPAC cycles were high, major complications were rare, and PRGS-defined histologic regression occurred in roughly half of evaluable patients, despite many being heavily pretreated and medically frail [32,33,36,37,38,56,57,58,59]. Earlier PDAC-specific PIPAC experience reported by Khosrawipour et al. also demonstrated feasibility and biological activity in heavily pretreated patients. In their 20-patient cohort treated with cisplatin–doxorubicin PIPAC, 35% of evaluable patients achieved objective histologic regression (TRG 2–4) and median OS was approximately 9 months, despite all patients having progressed on modern systemic regimens. Importantly, major adverse events were rare and no CTCAE grade ≥ 3 toxicities were observed, underscoring the safety of PIPAC even in a salvage setting. While the study predates PRGS standardization and used TRG-based pathology, its findings are consistent with later PIPAC series and reinforce the biologic plausibility of aerosolized intraperitoneal therapy in PDAC peritoneal metastases (60). Median OS of 9–13 months in this context compares favorably with best supportive care and approximates survival seen in contemporary second-line systemic trials for advanced PDAC [3,18,56,57,58,59,60,70].

The ability to reassess disease status laparoscopically, obtain serial biopsies for PRGS, and perform molecular profiling of peritoneal deposits or lavage fluid makes PIPAC particularly attractive as a translational platform [24,25,26,27,32,33,36,37,38,68,72]. Avula et al. and others argue that PIPAC may be uniquely positioned to test targeted or immunomodulatory agents aimed at the molecular mediators of peritoneal metastasis, including integrins, adhesion molecules, and immune checkpoints [6,24,25,39,67,71]. Early data from GI PIPAC–immunotherapy combinations, such as the PIANO trial in gastric cancer peritoneal metastases, provide proof of principle that regional chemotherapy can sensitize the peritoneal microenvironment to systemic immunotherapy; analogous strategies in PDAC PM are conceptually appealing but remain to be tested [68,70,71,72].

However, PIPAC is not without limitations. Many patients discontinue treatment after one or two cycles due to systemic progression, declining performance status, or logistical barriers [56,57,58,59,69,70]. Moreover, the encouraging disease stabilization and histologic regression reported in selected PIPAC cohorts likely reflect both treatment effect and inherently favorable tumor biology in patients able to undergo repeated cycles, and therefore should be interpreted cautiously rather than as evidence of a direct causal survival advantage. The optimal drug choice (oxaliplatin vs. cisplatin/doxorubicin vs. novel agents), dose intensities, and cycle frequency remain unsettled, and current protocols are largely extrapolated from colorectal and gastric experience rather than PDAC-specific pharmacology [24,25,26,27,56,57,58,59,69,70,71,73]. Additionally, standardized thresholds for defining meaningful PRGS response and for triggering escalation to CRS + HIPEC or conversion surgery are lacking. Larger prospective registries and harmonization initiatives will be required to move PIPAC from a promising niche intervention to an integrated pillar of PDAC PM management.

### 4.6. Biological Rationale and Emerging Translational Insights

The biological plausibility of IP strategies in PDAC is reinforced by growing mechanistic understanding of peritoneal metastasis. Avula et al. describe a stepwise peritoneal metastatic cascade involving tumor cell detachment, survival in peritoneal fluid, adhesion to mesothelium, invasion of the submesothelial stroma, and formation of vascularized implants, each step driven by specific molecular mediators such as integrins, adhesion molecules, matrix metalloproteinases, chemokine receptors, and immune-modulatory pathways [6,39,40,72]. Many of these processes are spatially confined to the peritoneal compartment, suggesting that maximizing local drug concentrations could disproportionately disrupt peritoneal progression relative to distant organ metastases.

Moreover, IP strategies may partially overcome the desmoplastic stromal barrier and hypovascularity that limit systemic therapy penetration into peritoneal deposits [6,7,8,39,40,41,72]. Hyperthermia enhances drug diffusion, impairs DNA repair, and alters stromal architecture, while high interperitoneal concentrations of paclitaxel or platinum agents can saturate the tumor–stroma interface, producing steep local concentration gradients that are difficult to achieve via systemic delivery alone [12,14,15,16,18,19,20,23,68,69,70,71,72,73]. Translational PIPAC studies have documented increased T-cell infiltration, modulation of immunosuppressive cell subsets, and dynamic changes in stromal markers after repeated cycles, providing early evidence that regional chemotherapy can “re-condition” the peritoneal microenvironment in ways that might synergize with modern immuno- and targeted therapies [24,25,26,27,33,36,37,38,68,72].

### 4.7. Patient Selection, Staging, and Integration into PDAC Care Pathways

A recurring theme across all modalities is the critical importance of meticulous patient selection. Wu et al. propose a structured management framework for PDAC peritoneal metastasis that integrates staging laparoscopy, peritoneal cytology, PCI scoring, and molecular profiling to triage patients toward systemic-only therapy, NIPEC/IP-PTX, PIPAC, CRS + HIPEC, or best supportive care [69,72]. Our review supports several pragmatic selection principles consistent with this framework:

Performance status and systemic disease control—IP strategies are best suited to patients with ECOG 0–1 who have at least stable disease or better on modern systemic regimens (e.g., FOLFIRINOX or GnP), as reflected in all CRS + HIPEC and NIPEC/IP-PTX series [44,45,46,47,48,49,50,51,52,53,54,55,69,70,71,73].

Peritoneal disease burden—Low to moderate PCI, absence of bulky non-peritoneal metastases, and radiologic or laparoscopic evidence of limited small-bowel serosal involvement appear critical prerequisites for CRS + HIPEC and conversion surgery [47,48,49,50,64,69,70,71,73].

Biologic risk markers—Emerging data on peritoneal cytology, ctDNA, and molecular profiling of peritoneal deposits suggest that biologic aggressiveness, rather than anatomic extent alone, may ultimately guide selection for aggressive locoregional strategies [10,39,40,41,70,72,73]. Beyond simply acknowledging their relevance, these biomarkers can be operationalized to guide treatment allocation. Patients with rapidly progressive systemic disease, persistently rising ctDNA, highly aggressive transcriptional phenotypes, or worsening PCI despite therapy likely represent “exclusion biology” in whom intraperitoneal therapy is unlikely to meaningfully alter trajectory. Conversely, patients demonstrating durable systemic disease control, favorable molecular biology, suppressed or declining ctDNA, and stable or improving PCI may represent potential “intraperitoneal responders”. Dynamic biomarkers may also inform treatment escalation: improving PRGS on sequential laparoscopic sampling, stable or falling ctDNA, and controlled PCI evolution could justify continuation of NIPEC/PIPAC or consideration of CRS + HIPEC in carefully selected patients, whereas biologic deterioration should prompt de-escalation. Incorporating such biology-based decision points will be crucial to transition PDAC peritoneal treatment strategies from anatomy-driven selection toward precision-guided therapy.

Treatment goals and patient preference—For patients prioritizing symptom control (e.g., refractory ascites) over aggressive cytoreduction, NIPEC/IP-PTX or PIPAC may offer meaningful palliation and disease stabilization without the morbidity of CRS + HIPEC [51,52,53,54,55,56,57,58,59,60,69,70,71,73].

Taken together, the encouraging outcomes reported across CRS + HIPEC, NIPEC/IP-PTX, and PIPAC cohorts should not be interpreted as universally applicable. Instead, these results most likely reflect a biologically favorable subset of patients with indolent disease kinetics, demonstrable systemic chemosensitivity, and the physiological reserve to tolerate aggressive multimodality therapy. This reinforces the principle that patient selection and underlying tumor biology remain the dominant determinants of benefit.

Moving forward, the integration of IP therapy into PDAC care pathways will likely depend on the development of consensus algorithms, similar to those now adopted for colorectal and gastric peritoneal metastases [12,13,17,71,73]. Such algorithms should incorporate not only oncologic endpoints but also patient-reported outcomes, quality of life, and healthcare resource utilization, particularly as many IP regimens are resource-intensive and concentrated in high-volume centers.

### 4.8. Contextualizing Intraperitoneal Strategies Against Contemporary Systemic Therapy Benchmarks

Although historical reports frequently cite survival of 3–6 months for PDAC with peritoneal metastases, contemporary systemic therapy has meaningfully improved outcomes in selected patients with good performance status. Modern regimens such as modified FOLFIRINOX and gemcitabine/nab-paclitaxel achieve median overall survival (OS) of 11–14 months in metastatic PDAC, with some series reporting ≥18 months in biologically favorable, chemotherapy-responsive subsets [74,75]. Importantly, recent analyses suggest that even among patients with peritoneal metastases, outcomes may approach 9–12 months with optimized systemic therapy alone in appropriately selected individuals, underscoring the prognostic impact of chemosensitivity and disease biology rather than anatomic disease distribution alone [47,48,59].

Accordingly, the interpretation of CRS + HIPEC, NIPEC/IP-PTX, and PIPAC outcomes must be framed against these modern systemic benchmarks rather than historical natural-history data. Survival signals approaching 14–30 months in IP cohorts likely reflect an interaction between treatment effect, favorable tumor biology, durable systemic response, and stringent patient selection rather than a purely modality-driven benefit. To contextualize intraperitoneal strategies against modern systemic therapy outcomes, Table 5 summarizes contemporary survival expectations across metastatic PDAC settings, including general metastatic disease, peritoneal metastases, chemotherapy-responsive subsets, and IP therapy cohorts, drawing upon landmark FOLFIRINOX and nab-paclitaxel/gemcitabine trials as well as recent PDAC-PM series [47,48,59,74,75].

### 4.9. Standardization of Outcomes and Reporting Framework

A major barrier to synthesizing evidence across HIPEC, NIPEC/IP-PTX, and PIPAC studies is the lack of uniformity in endpoint definitions. Across published cohorts, OS, DFS, and PFS are variably reported from peritoneal metastasis (PM) diagnosis, from initiation of intraperitoneal therapy, or from surgical intervention, substantially limiting cross-study comparability. To facilitate meaningful interpretation of future PDAC intraperitoneal therapy trials, we propose a minimum standardized endpoint set:

1. Overall survival (OS)
  - OS from time of PM diagnosis, to capture true disease trajectory
  - OS from initiation of intraperitoneal therapy, to enable between-modality comparisons
2. Progression-free survival (PFS)
  - Defined using harmonized radiologic and/or laparoscopic criteria
  - With prespecified handling of systemic vs. peritoneal progression
3. Disease control assessment
  - Routine reporting of Peritoneal Regression Grading Score (PRGS) for histologic response
  - Baseline PCI and dynamic PCI change where feasible
4. Morbidity and feasibility
  - Standardized Clavien–Dindo morbidity
  - Procedural completion rates and reasons for discontinuation
5. Patient-centered outcomes
  - Formal reporting of ascites control, symptom burden, and quality-of-life metrics

Uniform adoption of these metrics would transform the field from small, heterogeneous feasibility cohorts toward data that can support meta-analysis, rational patient selection, and true comparative effectiveness research.

### 4.10. Quality of Life, Treatment Burden, and Patient-Centered Outcomes

Although feasibility, safety, and survival are the dominant endpoints reported in PDAC intraperitoneal therapy studies, quality of life (QoL) considerations are equally critical—particularly because many NIPEC/IP-PTX and PIPAC indications are palliative or aimed at prolonged disease control rather than cure. Across published series, symptomatic benefits most consistently reported include ascites control, improved performance tolerance, and reduced need for repeated paracenteses, which may meaningfully affect daily functioning and comfort. However, formal QoL reporting remains sparse, and structured patient-reported outcome (PRO) tools are rarely incorporated [51,52,53,54,55,56,57,58,59,60,69,70,71,73].

Treatment burden must also be acknowledged. NIPEC/IP-PTX requires maintenance of an intraperitoneal access port and repeated infusion cycles, which can lead to device complications, infection, hematologic toxicities, and treatment fatigue. Similarly, although PIPAC is generally well tolerated, it requires repeated laparoscopic interventions and many patients discontinue therapy early due to systemic progression or functional decline [56,57,58,59,69,70,71]. Thus, benefit–toxicity balance should be assessed not only through objective response or survival but also in terms of patient-perceived value and treatment sustainability.

Going forward, we advocate that future PDAC intraperitoneal therapy trials systematically incorporate validated QoL instruments (e.g., EORTC QLQ-C30, PAN26 where feasible), standardized documentation of ascites-related symptom burden and functional outcomes, prospective assessment of treatment burden and early discontinuation, and formal integration of patient-reported outcomes into primary or secondary endpoints. Such patient-centered reporting is essential to determine whether biologic or radiologic signals of activity translate into meaningful real-world benefit for patients.

### 4.11. Limitations of the Current Evidence and Future Directions

This review has several limitations inherent to the underlying literature. Most included studies are small, single-arm, and non-randomized, with heterogeneous patient populations, regimens, and endpoints, precluding formal meta-analysis and limiting causal inference. Selection bias is pervasive—especially in CRS + HIPEC cohorts—and confounding by indication cannot be fully corrected, even in matched comparisons [47,48,49,50,64,69,73]. Survival endpoints are variably defined (from pancreatectomy, from PM diagnosis, or from initiation of IP therapy), and reporting of PCI, PRGS, toxicity, and quality-of-life metrics is inconsistent across studies [44,45,46,47,48,49,50,51,52,53,54,55,56,57,58,59,61,69,70,71,72,73]. Additionally, our review focused on published full-text articles, so ongoing or negative trials identified in registries may be underrepresented, raising the possibility of residual publication bias [42,43,69,70,71]. Furthermore, true incidence and biology of peritoneal metastases remain incompletely characterized, as PM is often radiologically occult, underdiagnosed, and inconsistently captured in clinical trials; robust molecular and clinical biomarkers capable of identifying patients most likely to benefit from locoregional approaches are still lacking [48,58,69,73].

Despite these constraints, the convergence of findings across independent cohorts, modalities, and geographic regions is striking. Adjuvant HIPEC appears capable of reducing LRR; CRS + HIPEC for low-volume PM can yield multi-year survival in selected patients; NIPEC/IP-PTX provides reproducible disease control, ascites palliation, and meaningful conversion-to-resection; and PIPAC offers a safe, repeatable option for frailer patients with evidence of histologic response. Recent systematic reviews focused specifically on PDAC, along with broader peritoneal metastasis literature, reinforce these impressions and call for rigorously designed phase II and III trials to validate these signals [69,70,71,72,73].

Future research priorities include: (1) multi-institutional prospective registries with standardized PCI, PRGS, and outcome reporting; (2) biomarker-driven trials incorporating ctDNA, peritoneal cytology, and molecular profiling into selection criteria; (3) randomized or carefully controlled studies comparing systemic-only regimens with combined systemic plus IP strategies in well-defined PDAC PM subsets; and (4) translational programs leveraging PIPAC and NIPEC/IP-PTX as platforms to test novel agents and immunomodulatory combinations [39,40,41,68,69,70,71,72,73]. Only through such coordinated efforts can the promise of intraperitoneal therapies be translated into robust, guideline-level recommendations for patients with PDAC and peritoneal involvement.

## 5. Conclusions

Intraperitoneal therapies represent a promising and increasingly refined set of strategies for addressing the significant unmet need posed by peritoneal recurrence and metastasis in pancreatic ductal adenocarcinoma. Across HIPEC, NIPEC/IP-paclitaxel, and PIPAC, available evidence demonstrates consistent feasibility, acceptable safety, and meaningful biological activity in carefully selected patients. Adjuvant HIPEC may reduce locoregional recurrence without adding perioperative risk; CRS plus HIPEC can yield multi-year survival in patients with limited peritoneal disease who respond well to systemic therapy; NIPEC/IP-paclitaxel offers reproducible ascites control and conversion-to-resection opportunities; and PIPAC provides a minimally invasive, repeatable option with objective histologic responses in patients with advanced disease. Collectively, these modalities achieve outcomes that exceed historical expectations for peritoneal involvement managed with systemic therapy alone.

However, the current evidence base remains constrained by small sample sizes, heterogeneous methodologies, and limited randomized data. Standardization of patient selection, staging, treatment protocols, and outcome measures is essential to clarify the true therapeutic value of each approach. Future efforts should prioritize prospective, biomarker-driven trials, integration with modern systemic regimens, and collaborative multicenter registries to refine indications and optimize sequencing. As understanding of peritoneal biology and pharmacologic delivery continues to advance, intraperitoneal therapy has the potential to become an important component of personalized, multimodal care in pancreatic cancer, offering select patients a realistic path to improved survival and quality of life.

## Figures and Tables

**Table 1:** Showing study characteristics.

Study	Year	Country	Modality	Setting	Design	*N*	Regimen	Systemic Therapy Backbone
Yurttas [44]	2021	Germany	HIPEC	Adjuvant	Phase I/II	16	Gem 1000 mg/m^2^, 42 °C, 60 min	Standard adjuvant
Padilla-Valverde [45]	2024	Spain	HIPEC	Adjuvant	RCT	42	Gem 120 mg/m^2^, 41–42 °C, 30 min	Gemcitabine
Padilla-Valverde [46]	2021	Spain	HIPEC	Adjuvant	Pilot	10	Gem 120 mg/m^2^ HIPEC	Gemcitabine
Grotz [47]	2023	USA	CRS + HIPEC	PM	Prospective	18	Cisplatin + MMC	Pre-op systemic (≥6 mo)
Gudmundsdottir [48]	2023	Iceland	CRS + HIPEC	PM	Retrospective	23	Institutional HIPEC	Chemo matched cohort
Yan [49]	2024	China	CRS + HIPEC	PM	Retrospective	10	HIPEC	Gem-based
Tentes [50]	2018	Greece	CRS + HIPEC	PM	Retrospective	23	HIPEC	Mixed
Satoi (Multicenter) [51]	2017	Japan	NIPEC	PM	Phase II	33	IP PTX 20 mg/m^2^ + IV PTX 50 mg/m^2^ + S-1	S-1
Satoi (Single center) [52]	2017	Japan	NIPEC	PM	Retrospective	20	IP PTX + S-1	S-1
Takahara [53]	2014	Japan	NIPEC	PM	Phase II	10	IP PTX + IV PTX + S-1	S-1
Yamada [54]	2020	Japan	NIPEC	PM	Phase I/II	46	IP PTX + GnP	GnP
Takahara [55]	2021	Japan	NIPEC	PM	Phase I	12	IP PTX + GnP	GnP
Horvath [56]	2018	Germany	PIPAC	PM	Retrospective	6	PIPAC CD/OX	Prior systemic
Kurtz [57]	2018	Denmark	PIPAC	PM	Prospective	7	PIPAC-OX	Outpatient
Di Giorgio [58]	2020	Italy	PIPAC	PM	Retrospective	14	PIPAC CD/OX	Mixed
Kim [59]	2021	USA	PIPAC	PM	Phase I	16	OX 45: 120 mg/m^2^	Prior chemo
Khosrawipour [60]	2017	Germany	PIPAC	PM	Retrospective	20	PIPAC CDDP/DOX	Prior systemic

**Table 2:** Showing Risk of Bias using the Newcastle–Ottawa Scale.

Study	Selection (0–4)	Comparability (0–2)	Outcome (0–3)	Total (0–9)
Yurttas [44]	4	1	3	8
Padilla-Valverde RCT [45]	4	2	3	9
Padilla-Valverde pilot [46]	3	1	3	7
Grotz [47]	4	2	3	9
Gudmundsdottir [48]	3	1	2	6
Yan [49]	3	1	2	6
Tentes [50]	2	1	2	5
Satoi multicenter [51]	3	1	3	7
Satoi single center [52]	3	1	2	6
Takahara 2014 [53]	3	1	2	6
Yamada [54]	4	1	3	8
Takahara 2021 [55]	4	1	3	8
Horvath [56]	3	1	2	6
Kurtz [57]	3	1	2	6
Di Giorgio [58]	3	1	2	6
Kim [59]	4	1	3	8
Khosrawipour [60]	3	1	2	6

**Table 3:** Showing results of individual study with extractable data.

Study	Feasibility	30 d Mortality	Major Complications	Response/PRGS	Conversion	mOS
Yurttas [44]	89%	0%	Comparable to pancreatectomy	–	–	16.1 mo
Padilla-Valverde (RCT) [45]	>90%	0–5%	Acceptable	Decreased LRR	–	~17–18 mo
Grotz [47]	High	0%	20–43%	–	–	26 mo
Gudmundsdottir [48]	High	<5%	Acceptable	–	–	41 mo
Yan [49]	High	0%	Low	–	–	24.2 mo
Satoi multicenter [51]	High	0%	Manageable	–	24%	16.3 mo
Yamada [54]	High	–	–	–	17%	14.5 mo
Di Giorgio [58]	High	0%	Mostly G1–2	50% PRGS regression	–	9.7 mo
Kim (PIPAC) [59]	High	–	Low	PRGS improvement	–	~12 mo
Khosrawipour [60]	High	~2%	Low (no CTCAE ≥ 3; 1 SBO death)	35% histologic regression (TRG)	-	~9 mo

**Table 4:** Showing Comparative Characteristics of Intraperitoneal Therapies for Pancreatic Cancer.

Modality	Ideal Patients	Strengths	Limitations	Safety Notes	Evidence Level
HIPEC (Adjuvant)	Resected PDAC; high LRR risk	Decrease in LRR; good safety; RCT data	Requires OR time; limited centers	Similar to major abdominal surgery	Moderate
CRS + HIPEC	Isolated PM; good response to chemo	Multi-year survival; CC-0/1 possible	Highly selected patients	Major morbidity 20–40%	Low-Moderate
NIPEC/IP-PTX	PM with ascites; unresectable	Ascites control; conversion surgery	Requires port; hematologic AEs	Mostly manageable	Low-Moderate
PIPAC	Heavily pretreated PM	Histologic regression; low toxicity	Early discontinuation in PD	Safe outpatient profile	Absent

**Table 5:** Showing Contemporary survival expectations across metastatic pancreatic ductal adenocarcinoma (PDAC) clinical settings, including systemic therapy benchmarks and outcomes reported with intraperitoneal (IP) treatment strategies.

Clinical Setting	Contemporary Expected Outcome	Representative Evidence
General metastatic PDAC	11–14 months median OS with modern systemic therapy	Conroy et al., NEJM 2011; Von Hoff et al., NEJM 2013 [74,75]
PDAC with peritoneal metastases (unselected)	Historically 3–6 months; contemporary selected cohorts ~9–12 months	Wu et al. 2025; Gudmundsdottir 2023 [48,69]
Selected PM patients with chemotherapy-responsive biology	≥12–18 months reported in modern cohorts	Gudmundsdottir 2023; Grotz 2023 [47,48]
IP strategies (HIPEC/NIPEC/PIPAC)	14–30 months in highly selected series	Yurttas 2021; Padilla-Valverde 2024; Satoi; Yamada; Di Giorgio; Robella; Choi [44,45,51,54,58,63]

## Data Availability

All data analyzed in this review are included within published studies.

## Supplementary Materials

The following supporting information can be downloaded at: https://www.mdpi.com/article/10.3390/cancers18020182/s1. Table S1: PRISMA 2020 checklist.

## Abbreviations

The following abbreviations are used in this manuscript:

AE	Adverse event
CC-0/1	Complete or near-complete cytoreduction (no visible disease/residual disease < 2.5 mm)
CRS	Cytoreductive surgery
DFS	Disease-free survival
GnP	Gemcitabine plus nab-paclitaxel
HIPEC	Hyperthermic intraperitoneal chemotherapy
IP	Intraperitoneal
LRR	Locoregional recurrence
mOS	Median overall survival
NIPEC	Normothermic intraperitoneal chemotherapy
IP-PTX/NIPEC-PTX	Intraperitoneal paclitaxel
OR	Operating room
OS	Overall survival
PCI	Peritoneal carcinomatosis index
PD	Progressive disease
PDAC	Pancreatic ductal adenocarcinoma
PFS	Progression-free survival
PIPAC	Pressurized intraperitoneal aerosol chemotherapy
PM	Peritoneal metastases
PRGS	Peritoneal Regression Grading Score
RCT	Randomized controlled trial
TME	Tumor microenvironment
