# Supplementary material for: Intraperitoneal Chemotherapy Strategies in Pancreatic Ductal Adenocarcinoma: A Systematic Review of Hyperthermic Intraperitoneal Chemotherapy, Normothermic Intraperitoneal Chemotherapy, and Pressurized Intraperitoneal Aerosol Chemotherapy
Source: Cancers (Basel). 2026 Jan 6;18(2):182. doi: 10.3390/cancers18020182 (PMC12838573; doi:10.3390/cancers18020182)
Supplement: Supplementary file 1 [file cancers-18-00182-s001.zip › cancers-4055957-supplementary.pdf]

Table S1. PRISMA 2020 Checklist (Completed)

Section / Topic	Item #	Checklist Item	
Title	1	Identify the report as a systematic review.	✓
Abstract	2	Provide a structured summary.	✓
Introduction	3	Describe rationale.	✓
Introduction	4	Provide objectives.	✓
Methods	5	Eligibility criteria.	✓
Methods	6	Information sources.	✓
Methods	7	Search strategy.	✓
Methods	8	Selection process.	✓
Methods	9	Data collection process.	✓
Methods	10	Data items.	✓
Methods	11	Study risk of bias assessment.	✓
Methods	12	Effect measures.	✓
Methods	13	Synthesis methods.	✓
Results	14	Study selection.	✓
Results	15	Study characteristics.	✓
Results	16	Risk of bias in studies.	✓
Results	17	Results of individual studies.	✓

Results	18	Synthesis results.	✓
Discussion	19	Summary of main findings.	✓
Discussion	20	Limitations.	✓
Discussion	21	Conclusions.	✓
Other	22	Registration, protocol availability.	✓
Other	23	Support, competing interests.	✓
